# Association of pulse pressure with hematoma expansion in patients with spontaneous supratentorial intracerebral hemorrhage

**DOI:** 10.3389/fneur.2024.1374198

**Published:** 2024-05-15

**Authors:** Chao-Ying Wang, Su-Zhen Lai, Bao-Cai Kang, Yi-Zhao Lin, Chun-Juan Cao, Xin-Bing Huang, Jian-Qun Wang

**Affiliations:** ^1^Department of Neurosurgery, Dehua County Hospital, Quanzhou, China; ^2^Department of Imaging, Dehua County Hospital, Quanzhou, China; ^3^Department of Internal Medicine, Dehua County Hospital, Quanzhou, China; ^4^Department of Geriatrics, Changji People’s Hospital, Changji, China; ^5^Department of Laboratory Medicine, Dehua County Hospital, Quanzhou, China; ^6^Department of Neurology, Dehua County Hospital, Quanzhou, China

**Keywords:** stroke, cerebral hemorrhage, blood pressure, hematoma growth, predictor, dose–response relationship

## Abstract

**Objective:**

Recent reports have demonstrated that a wider pulse pressure upon admission is correlated with heightened in-hospital mortality following spontaneous supratentorial intracerebral hemorrhage (ssICH). However, the underlying mechanism remains ambiguous. We investigated whether a wider pulse pressure was associated with hematoma expansion (HE).

**Methods:**

Demographic information, clinical features, and functional outcomes of patients diagnosed with ssICH were retrospectively collected and analyzed. Multivariate logistic regression was conducted to identify independent predictors of HE. Weighted logistic regression, restricted cubic spline models, and propensity score matching (PSM) were employed to estimate the association between pulse pressure and HE.

**Results:**

We included 234 eligible adult ssICH patients aged 60 (51–71) years, and 55.56% were male. The mean pulse pressure was 80.94 ± 23.32 mmHg. Twenty-seven patients (11.54%) developed early HE events, and 116 (49.57%) experienced a poor outcome (modified Rankin scale 3–6). A wider mean pulse pressure as a continuous variable was a predictor of HE [odds ratios (OR) 1.026, 95% confidence interval (CI) 1.007–1.046, *p* = 0.008] in multivariate analysis. We transformed pulse pressure into a dichotomous variable based on its cutoff value. After adjusting for confounding of HE variables, the occurrence of HE in patients with ssICH with wider pulse pressure levels (≥98 mmHg) had 3.78 times (OR 95% CI 1.47–9.68, *p* = 0.006) compared to those with narrower pulse pressure levels (<98 mmHg). A linear association was observed between pulse pressure and increased HE risk (*P* for overall = 0.036, *P* for nonlinear = 0.759). After 1:1 PSM (pulse pressure ≥98 mmHg vs. pulse pressure <98 mmHg), the rates of HE events and poor outcome still had statistically significant in wider-pulse pressure group [HE, 12/51 (23.53%) vs. 4/51 [7.84%], *p* = 0.029; poor outcome, 34/51 (66.67%) vs. 19/51 (37.25%), *p* = 0.003].

**Conclusion:**

Widened acute pulse pressure (≥98 mmHg) levels at admission are associated with increased risks of early HE and unfavorable outcomes in patients with ssICH.

## Introduction

Spontaneous supratentorial intracerebral hemorrhage (ssICH) is one of the most catastrophic types of strokes with high morbidity, disability, and mortality, and poses a substantial burden on health and social services (1). Most brain injuries result from the mechanical effects of the initial hemorrhage (2). Nonetheless, hematoma expansion (HE) contributes to secondary brain damage and is a major determinant of clinical deterioration and unfavorable prognosis (3–5).

After the onset of symptoms of intracerebral hemorrhage (ICH), there is often a sudden rise in blood pressure (BP), which is associated with an increased risk of hematoma growth and a poor prognosis (6). Numerous studies have linked high BP with HE and adverse clinical outcomes (4, 6, 7). The VISTA-ICH Cohort finding indicates that elevated blood pressure within 24 h after ICH is associated with HE and unfavorable prognosis (8). Several subsequent studies have revealed that acutely elevated systolic blood pressure (SBP) is independently associated with HE following spontaneous intracerebral hemorrhage (sICH) (9, 10). A meta-analysis of individual patient data from randomized clinical trials has demonstrated that intensive SBP lowering is safe and significantly reduces hematoma growth, implicating also that elevated SBP may be associated with early hematoma enlargement (11).

However, current guidelines for defining and treating spontaneous cerebral hemorrhage focus on SBP and diastolic blood pressure (DBP) rather than pulse pressure (12). Pulse pressure is defined as the difference between SBP and DBP, reflecting the stiffness of the large arteries (13). Pulse pressure increases with age and may be a key indicator of blood pressure in the elderly (14). According to the strain vessel hypothesis, small, short, penetrating arteries in the brain arising from large arteries are exposed to high pressures and maintain high vascular tone, thus providing a large pressure gradient from the large arteries to the penetrating arteries (15). As arterial stiffness increases, pulse wave velocity increases (16). A study has revealed that increased arterial stiffness is physiologically associated with the development of cerebral small vessel disease, such as cerebral microhemorrhage (16, 17). Therefore, in stroke patients with cerebral microbleeds or cerebral hemorrhages, increased pulse pressure may lead to increased perforating arterial injury.

Secondary analyses of the Antihypertensive Treatment of Acute Cerebral Hemorrhage II study (ATACH2) trial demonstrated that the time-sensitivity of medical interventions targeting HE, in which rapid intensive blood pressure lowering (within 2 h of the onset of ICH symptoms) reduced HE and improved functional outcomes (6). Indeed, the complex relationships between blood pressure parameters and clinical characteristics in patients with ICH have not been thoroughly investigated. Recent literature demonstrates that wide pulse pressure is an independent risk factor and a predictor of in-hospital mortality (18). Nonetheless, the role of pulse pressure in ICH patients is not fully understood, and its potential to differentiate risks and predict early HE has not been well-explored. Hence, this study investigated the correlation between admission pulse pressure and early HE in patients with ssICH.

## Methods and materials

### Study design and population

**Figure 1:**
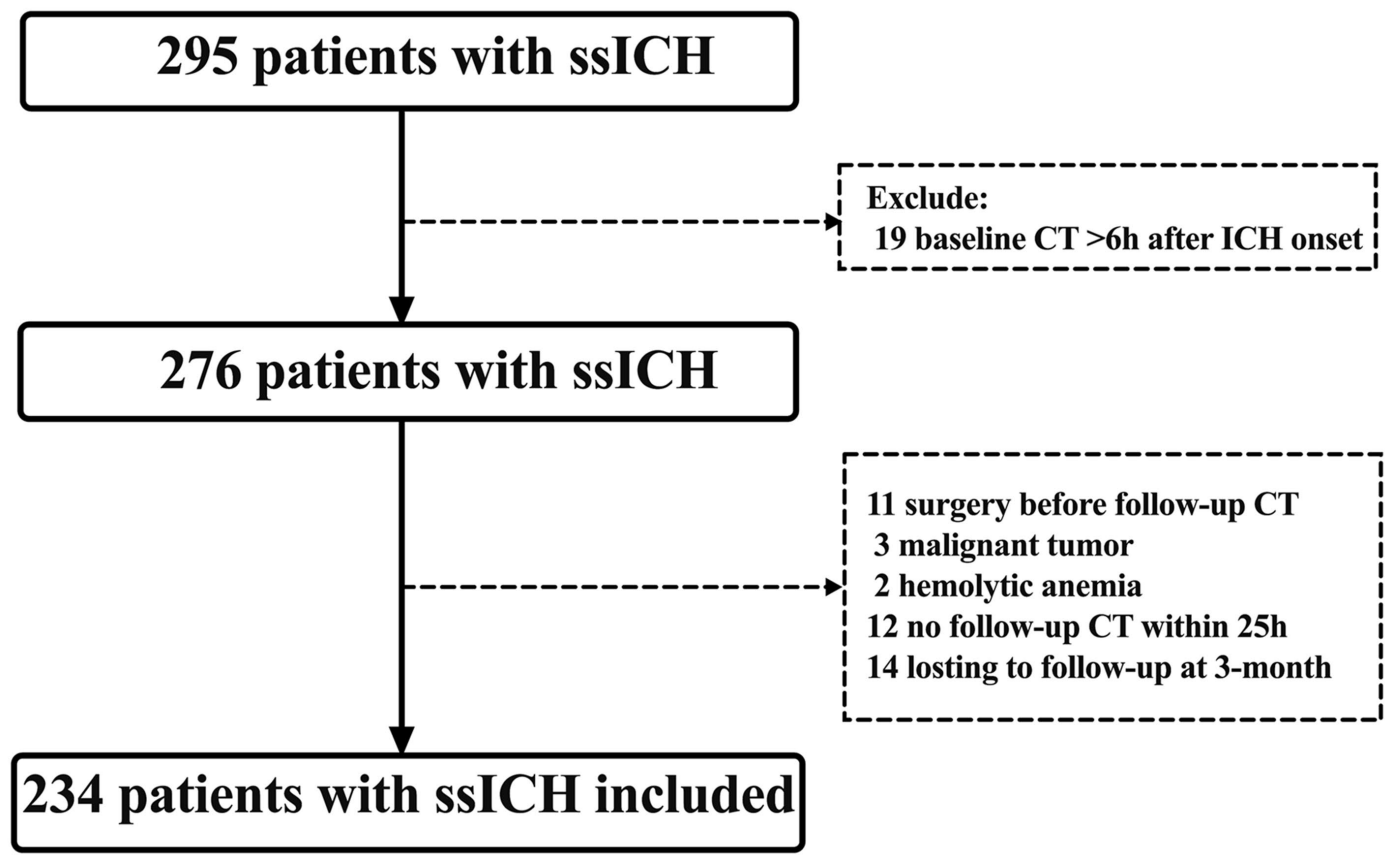
Flowchart screening for eligibility. CT, computed tomography; ICH, intracerebral hemorrhage; ssICH, spontaneous supratentorial intracerebral hemorrhage.

This retrospective observational study was conducted at Dehua County Hospital for Neurology and Neurosurgery between December 2020 and July 2023, focusing on patients aged 20 years and older who diagnosed with ssICH. The ethical clearance was obtained from the institutional ethical committee. Patients who met the following inclusion criteria were considered eligible: (a) they had confirmed initial supratentorial ICH by computed tomography (CT) scan within 6 h of symptom onset; (b) they underwent follow-up CT scans within 25 h after the baseline CT scan (19). Exclusion criteria included: (a) age < 20 years old; (b) having incomplete or missing case data; (c) undergoing emergency surgery before follow-up CT; (d) having a malignant tumor or hemolytic anemia. A flow diagram of the patient selection process is presented in Figure 1.

Clinical management followed the American Heart Association and American Stroke Association guidelines (12). Patients with stable neurological status or those who were unsuitable for surgery due to age or comorbidities were given conservative treatment. When the ICH volume exceeded 30 mL, or the patient’s consciousness declined, a craniotomy to remove the hematoma or cranial decompression resection was performed (12). Concomitant external ventricular drainage was performed if acute hydrocephalus or elevated intracranial pressure occurred.

### Data collection

Baseline characteristics and clinical information were collected from the electronic database. Baseline characteristics included demographics (age, sex), medical history (hypertension and diabetes mellitus), adverse lifestyle factors (smoking and alcoholism), admission SBP, DBP, admission Glasgow Coma Scale (GCS) score, time from onset of illness to baseline CT, ssICH location, baseline ssICH volume, presence of intraventricular hemorrhage (IVH), treatment, and admission laboratory parameters. Admission laboratory parameters included hemoglobin, platelets, activated partial thromboplastin time (aPTT), thrombin time (PT), and international normalized ratio (INR). Treatment options for surgery included microinvasive removal of hematoma, craniotomy evacuation of hematoma, and decompressive craniectomy.

### Blood pressure variables

Upon arrival at the hospital, the on-admission BP was recorded immediately. BP was measured according to the standard in-hospital care protocol, an automated, non-invasive cuff measurement of systolic and diastolic blood pressure at the mid-biceps. Mean arterial pressure (MAP) was calculated by the formula (SBP + 2 × DBP)/3. Pulse pressure was determined by the absolute difference between SBP and DBP (19). An intensive strategy to lower the patient’s SBP to 150–160 mmHg, especially in patients with SBP over 220 mmHg after ssICH (2). Close monitoring of renal function and renal volume status was necessary.

### Imaging analysis

The CT scan was conducted upon admission, and a subsequent CT scan was reviewed within 25 h. The 25 h cut-off was selected to encompass all CT scans performed within 24 h following the initial scan, typically done right away or within the first few minutes after admission (19). Assessment of the initial CT included determining the location of the hematoma (lobar or deep) and the presence of IVH. Supratentorial deep ICH is characterized by its selective involvement of the thalamus, basal ganglia, internal capsule, and deep periventricular white matter. At the same time, lobar ICH originates at the cortex and cortical–subcortical junction (20). Using a post-processing workstation (Advantage Workstation 4.6, GE Healthcare, Chicago, Illinois, United States), hematoma volumes on the initial and follow-up CT scans were estimated (21). HE was defined as an increase of hematoma volume >6 mL and relative hematoma volume increase >33% between initial and follow-up CT scans (5, 22).

### Outcome assessment

A favorable outcome is indicated by a modified Rankin scale (mRS) score <3 (mRS score 0–2, representing good recovery, no significant disability, or slight disability), while a poor outcome is indicated by an mRS score ≥3 (mRS score 3–6, representing moderate disability, moderately severe disability, severe disability, or death) (23). Outpatient clinic visits, telephone interviews, and WeChat interviews were utilized to evaluate the mRS grades three months post-discharge (24).

### Statistical analysis

For the statistical analyses, SPSS Statistics 26 (IBM, Armonk, NY), MedCalc version 18.2.1 (MedCalc Software, Ostend, Belgium), and R 4.2.1(RStudio, Boston, MA, United States) were all employed. A two-sided *p* < 0.05 was established as statistically significant. The mean ± standard deviation (SD), or median (interquartile range, IQR) was used to present continuous variables, while counts with percentages [*n* (%)] were used for categorical variables. Normal distribution was assessed by the Shapiro–Wilk normality test. Continuous variables were compared using the Student’s *t*-test (normal distribution) or the Mann–Whitney *U*-test (nonnormal distribution). Categorical variables were compared using the *χ*^2^ test or Fisher’s exact test. HE associates with an univariable *p*-value of less than 0.05 were included in the multivariate models. Multivariate logistic regression and linear analysis examined the independent association between pulse pressure and outcome variables.

Then, the area under a receiver-operating characteristic (ROC) curve was determined without covariate adjustment. Pulse pressure was transformed into a binary categorical variable based on the ROC analysis’s threshold to make the model better applied to clinical practice. Weighted multivariate linear regression models were developed. The pulse pressure was dichotomized as “< optimal cutoff value” and “≥ optimal cutoff value. Model I was unadjusted. Model II was adjusted for the variables with a *p*-value of less than 0.05 in univariable analysis.

The restricted cubic spline (RCS) model is a statistical analysis method used to create a smooth curve that visually describes the nonlinear relationship between pulse pressure and early HE. This study used the RCS model to observe nonlinear trends, with the 5th, 50th, and 95th pulse pressures serving as the three knots. Our construction of the RCS models was based on weighted multivariate linear regression models.

Moreover, the patients with ssICH were categorized into a wider pulse pressure group and a narrower group based on the optimal cutoff value obtained from ROC analysis. After conducting propensity score matching (PSM) with a 1:1 match and a caliper of 0.01 using the nearest neighbor approach to account for all potential confounders, the rates of early HE events, and poor outcomes were compared between the two groups.

## Results

### Baseline characteristics

After screening according to the inclusion and exclusion criteria, 234 adult patients with ssICH were included in this study (Figure 1). The Shapiro–Wilk normality test for continuous variables is displayed in Supplementary Table S1. Of the continuous variables, all continuous variables were non-normally distributed except for SBP, MAP, and pulse pressure. The median age of the included patients was 60.0 (IQR, 51.0–71.0) years and 55.56% were male. 82.05% of patients had a history of hypertension, indicating a significant prevalence among the study population. The mean SBP was 185.92 ± 30.21 mmHg, while the median DBP was 103 (IQR, 93.75–114) mmHg. The mean pulse pressure was 80.94 ± 23.32 mmHg. The median time from symptom onset to initial CT was 2 h (IQR, 1.0–3.0 h), the median baseline GCS scores being 14 (IQR, 9.0–15), and the median baseline volume was 8.4 mL (IQR, 4.12–14.0 mL). Out of the 234 patients, 27 patients (11.54%) developed early HE events, and 116 (49.57%) experienced a poor outcome (mRS 3–6). The baseline characteristics are summarized in detail in Table 1.

**Table 1:** Baseline characteristics for the 234 included ssICH patients.

Characteristics	Value
Age (years), median (IQR)	60.0 (51.0–71.0)
Sex	
Male, *n* (%)	130 (55.56)
Female, *n* (%)	104 (44.44)
Medical history, *n* (%)	
Hypertension, *n* (%)	192 (82.05)
Diabetes mellitus, *n* (%)	23 (9.83)
Smoking, *n* (%)	32 (13.68)
Alcoholism, *n* (%)	31 (13.25)
Admission blood pressure (mmHg)	
Systolic blood pressure, mean ± SD	185.92 ± 30.21
Diastolic blood pressure, median (IQR)	103 (93.75–114.0)
Mean arterial pressure, mean ± SD	131.96 ± 19.61
Pulse pressure, mean ± SD	80.94 ± 23.32
Time from symptom onset to initial CT (h), median (IQR)	2.00 (1.00–3.00)
Admission GCS score, median (IQR)	14.00 (9.00–15.00)
Baseline ssICH volume (mL), median (IQR)	8.40 (4.12–14.00)
Admission laboratory	
Hemoglobin (g/L), median (IQR)	137 (127–149)
Platelets, (×10^9^/L), median (IQR)	218.5 (181.75–263.0)
Prothrombin time (seconds), median (IQR)	11.3 (10.7–12.0)
International normalized ratio, median (IQR)	1.03 (0.97–1.09)
Activated partial thromboplastin time (seconds), median (IQR)	29.45 (26.80–32.70)
ssICH location, *n* (%)	
Deep	219 (93.59)
Lobar	15 (6.41)
Present of intraventricular hemorrhage, *n* (%)	86 (36.75)
Surgical treatment, *n* (%)	20 (8.55)
Hematoma expansion events, *n* (%)	27 (11.54)
mRS score, *n* (%)	
0–2	118 (50.43)
3–6	116 (49.57)

ssICH, spontaneous supratentorial intracerebral hemorrhage; mRS, modified Rankin scale; SD, standard deviation; IQR, interquartile range.

### Association of pulse pressure with early HE and poor outcome

Table 2 presents demographic characteristics, clinical features, and laboratory data that may have significance for HE. Univariate analysis revealed a statistically significant association between the occurrence of HE and SBP (*p* = 0.005), pulse pressure (*p* < 0.001), time from onset to baseline CT (*p* = 0.021), and admission GCS score (*p* = 0.005).

**Table 2:** Univariate analysis of patient characteristics associated with early hematoma expansion following ssICH.

Characteristics	Non-HE	HE	*p*-value
Age (years), median (IQR)	60.0 (52.0–71.0)	56.0 (47.5–68.5)	0.122
Sex			0.217
Male, *n* (%)	112 (54.11)	18 (66.67)	
Female, *n* (%)	95 (45.89)	9 (33.33)	
Medical history, *n* (%)			
Hypertension, *n* (%)	167 (80.68)	25 (92.59)	0.129
Diabetes mellitus, *n* (%)	18 (8.70)	5 (18.52)	0.107
Smoking, *n* (%)	26 (12.56)	6 (22.22)	0.169
Alcoholism, *n* (%)	29 (14.01)	2 (7.41)	0.314
Admission blood pressure (mmHg)			
Systolic blood pressure, mean ± SD	183.95 ± 30.29	201.07 ± 25.32	0.005
Diastolic blood pressure, median (IQR)	102.0 (93.0–114.0)	110.0 (100.0–113.0)	0.298
Mean arterial pressure, mean ± SD	131.21 ± 20.03	137.77 ± 15.10	0.102
Pulse pressure, mean ± SD	79.11 ± 22.85	94.96 ± 22.46	<0.001
Time from symptom onset to initial CT (h), median (IQR)	2.00 (1.00–3.00)	1.00 (1.00–2.00)	0.021
Admission GCS score, median (IQR)	14.00 (9.00–15.00)	10.00 (5.00–14.50)	0.005
Baseline ssICH volume (mL), median (IQR)	8.00 (4.00–14.00)	10.03 (7.00–12.09)	0.144
Admission laboratory			
Hemoglobin (g/L), median (IQR)	137.0 (127.0–149.0)	133.0 (128.0–148.0)	0.873
Platelets, (×10^9^/L), median (IQR)	218.0 (183.5–263.0)	219.0 (180.5–251.5)	0.977
Prothrombin time (seconds), median (IQR)	11.3 (10.7–12.0)	11.0 (10.4–11.7)	0.09
International normalized ratio, median (IQR)	1.03 (0.97–1.10)	1.01 (0.95–1.08)	0.219
Activated partial thromboplastin time (seconds), median (IQR)	29.30 (27.05–32.40)	29.70 (26.10–33.95)	0.597
ssICH location, *n* (%)			0.822
Deep	194 (93.72)	25 (92.59)	
Lobar	13 (6.28)	2 (7.41)	
Present of intraventricular hemorrhage, *n* (%)	78 (37.68)	8 (29.63)	0.414
mRS score, *n* (%)			0.002
0–2	112 (54.11)	6 (22.22)	
3–6	95 (45.89)	21 (77.78)	

ssICH, spontaneous supratentorial intracerebral hemorrhage; mRS, modified Rankin scale; SD, standard deviation; IQR, interquartile range.

**Figure 2:**
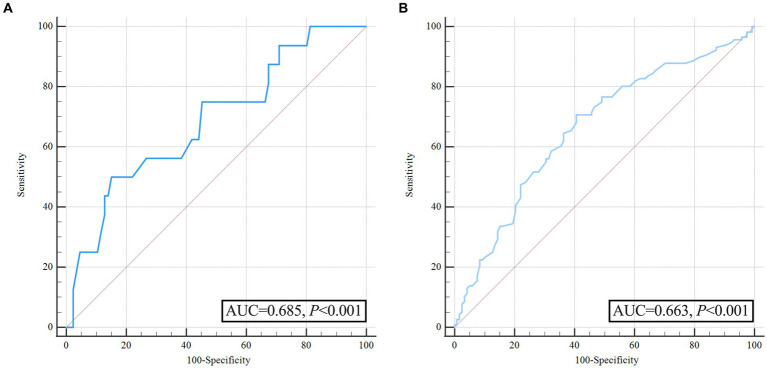
Receiver operating characteristic curve analysis of pulse pressure for predicting hematoma expansion and unfavorable outcome before propensity score matching. **(A)** ROC for pulse pressure to predict hematoma expansion; **(B)** ROC for pulse pressure to predict poor outcome.

A multivariate logistic regression analysis was conducted to avoid the confounding effects of each factor. Variables with significant effects in univariate analyses (*p* < 0.05), including admission GCS score, pulse pressure, and time from symptom onset to baseline CT, were assessed in multivariate analyses. The area under the curve (AUC) of pulse pressure and the AUC of SBP were comparable by *Z*-test, and the AUC of pulse pressure (AUC = 0.685) was slightly greater than that of SBP (AUC = 0.667). Still, no statistically significant difference existed (*Z* = 0.562, *p* = 0.574). In addition, there may be multicollinearity between pulse pressure and SBP. Finally, we referred to previous literature and included pulse pressure in the multivariable analysis instead of SBP (18, 19). Multivariable analysis revealed wider pulse pressure levels as a continuous variable were statistically associated with HE [pulse pressure, odds ratios (OR) 1.026, 95% confidence interval (CI) 1.007–1.046, *p* = 0.008, Table 2], while the time from illness onset to baseline CT (OR 0.629, 95%CI 0.394–1.004, *p* = 0.052), and admission GCS score (OR 0.91, 95%CI 0.82–1.011, *p* = 0.079) were not associated with HE. In Figure 2A and Table 3, the ROC analysis demonstrated an AUC of 0.685 (95% CI 0.622–0.744, *p* < 0.0001, Figure 2A), with a sensitivity of 48.15% and specificity of 80.19% for predicting HE in ssICH patients, and the optimal cutoff threshold of pulse pressure level was identified at 98 mmHg (Table 3). Moreover, we found that HE was associated with an increased risk of 3 month poor outcome (mRS 3–6, HE vs. Non-HE: 77.78% vs. 45.89%, *p* = 0.002, Table 2). ROC analysis of pulse pressure to predict poor outcome determined the best cut-off value of 78 mmHg with an AUC of 0.663 (95% CI, 0.598–0.723, *p* < 0.001, Figure 2B and Table 3).

**Table 3:** Area under the pulse pressure curve associated with early hematoma expansion and poor outcomes at 3 months.

	Cutoff value (mmHg)	AUC	95%CI of AUC	*p*-value	Sensitivity (%)	Specificity (%)	Youden’s index
Before PSM							
Early HE	98	0.685	0.622–0.744	<0.001	48.15	80.19	0.2834
Poor outcome	78	0.663	0.598–0.723	<0.001	70.69	59.32	0.3001
After PSM							
Early HE	111	0.686	0.587–0.774	0.013	50.0	84.88	0.3488
Poor outcome	91	0.683	0.583–0.771	<0.001	79.25	57.14	0.3639

HE, hematoma expansion; PSM, propensity score matching; AUC, area under the curve; CI, confidence interval.

We converted pulse pressure into a binary variable and developed several multivariate models to investigate the effect of different pulse pressure ranges on early HE. The pulse pressure was dichotomized as “<98 mmHg” and “≥98 mmHg.” Two weighted logistic regression models were used to analyze the relationship between pulse pressure and early HE in patients with ssICH, as presented in Table 4. We constructed a weighted logistic regression model, adjusting for variables with *p* < 0.05 in the univariate analysis (including admission GCS score and time from illness onset to initial CT), which revealed that HE occurred 3.78 times (95% CI 1.47–9.68, *p* = 0.006) more frequently in patients with pulse pressure ≥98 mmHg than in those with pulse pressure <98 mmHg. Therefore, the result of the regression model supports the conclusion that wider pulse pressure levels (≥98 mmHg) are associated with an increased risk of early HE occurrence in patients with ssICH.

**Table 4:** ORs (95% CIs) of the association between pulse pressure and early hematoma expansion in multivariate adjustment models.

	Model I		Model II	
	OR (95%CI)	*P* value	OR (95%CI)	*P* value
<98 mmHg	Reference		Reference	
≥98 mmHg	3.76 (1.64–8.61)	0.002	3.78 (1.47–9.68)	0.006

OR, odds ratios; CI, confidence interval. Model I was adjusted none. Model II was adjusted for admission GCS score and time from illness onset to initial CT.

### Dose–response relationship between pulse pressure level and the risk of early HE

**Figure 3:**
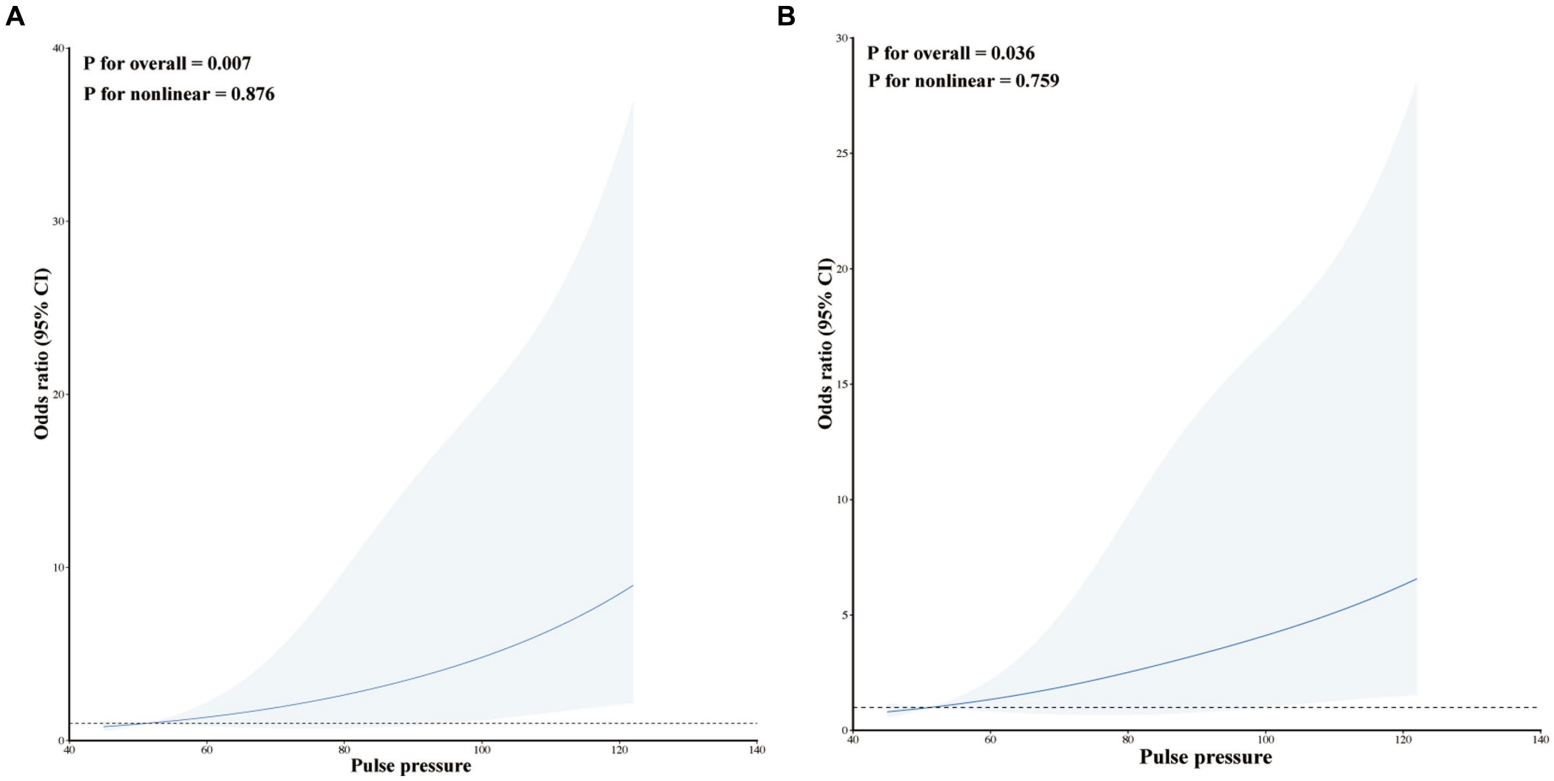
Restricted cubic spline modeling the relationship between pulse pressure and HE in patients with ssICH. **(A)** Model I for HE; **(B)** Model II for HE. Model I was adjusted none. Model II was adjusted for time from illness onset to initial CT and admission GCS score.

Our study utilized RCS plots to illustrate the relationships between pulse pressure levels and early HE. Two models were developed to adjust for different confounding factors. Results from two linear RCS models demonstrated a significant association between pulse pressure level and an increased risk of HE (model I, *p* for overall = 0.007, *p* for nonlinear = 0.876; model II, *p* for overall = 0.036, *p* for nonlinear = 0.759; Figures 3A,B). The RCS curve indicated that the risk of HE increased as pulse pressure increased (Figure 3).

### Propensity score matching

The patients with ssICH were categorized into a wider pulse pressure group (pulse pressure ≥ 98 mmHg, *n* = 180) and a narrower pulse pressure group (pulse pressure < 98 mmHg, *n* = 54) based on the optimal threshold. Univariate analysis revealed significant differences in history of hypertension, SBP, DBP, MAP, pulse pressure, admission GCS score, and early HE and mRS score between the two groups (Table 5).

**Table 5:** Characteristics of patients dichotomized to the identified pulse pressure threshold (98 mmHg) before and after propensity-score matching.

Characteristics	Before propensity-score matching			After propensity-score matching		
	<98 mmHg (*n* = 180)	≥98 mmHg (*n* = 54)	*p*-value	<98 mmHg (*n* = 51)	≥98mmhg (*n* = 51)	*p*-value
Age (years), median (IQR)	60.0 (51.0–70.0)	60.0 (50.0–74.5)	0.715	61.00 (53.00–70.00)	60.00 (50.00–74.00)	0.907
Sex			0.755			0.417
Male, *n* (%)	101 (56.11)	29 (53.70)		33 (64.71)	29 (56.86)	
Female, *n* (%)	79 (43.89)	25 (46.30)		18 (35.29)	22 (43.14)	
Medical history, *n* (%)						
Hypertension, *n* (%)	139 (77.22)	53 (98.15)	<0.001	50 (98.04)	50 (98.04)	1.00
Diabetes mellitus, *n* (%)	16 (8.89)	7 (12.96)	0.378	4 (7.84)	5 (9.80)	0.727
Smoking, *n* (%)	25 (13.89)	7 (12.96)	0.862	8 (15.69)	7 (13.73)	0.78
Alcoholism, *n* (%)	22 (12.22)	9 (16.67)	0.398	7 (13.73)	9 (17.65)	0.586
Admission blood pressure (mmHg)						
Systolic blood pressure, mean ± SD	175.53 ± 24.78	220.57 ± 18.67	<0.001	184.69 ± 21.33	220.80 ± 19.19	<0.001
Diastolic blood pressure, median (IQR)	102.0 (93.0–113.25)	109.0 (98.5–114.75)	0.03	106.00 (98.00–119.50)	110.00 (99.00–114.50)	0.644
Mean arterial pressure, mean ± SD	127.77 ± 18.53	145.94 ± 16.48	<0.001	133.39 ± 16.72	146.25 ± 16.80	<0.001
Time from symptom onset to initial CT (h), median (IQR)	2.00 (1.00–3.00)	2.00 (1.00–2.00)	0.72	2.00 (1.00–2.00)	2.00 (1.00–2.00)	0.751
Admission GCS score, median (IQR)	14.00 (9.00–15.00)	12.00 (7.00–15.00)	0.035	12.00 (7.00–15.00)	12.00 (7.00–15.00)	0.959
Baseline ssICH volume (mL), median (IQR)	8.00 (4.04–13.92)	10.00 (4.76–14.17)	0.449	8.00 (4.03–13.58)	10.00 (4.80–14.12)	0.484
Admission laboratory						
Hemoglobin (g/L), median (IQR)	138.0 (127.0–149.25)	135.0 (123.8–147.5)	0.374	136.00 (129.00–148.50)	135.00 (126.50–148.00)	0.618
Platelets, (×10^9^/L), median (IQR)	217.5 (181.75–267.5)	223.0 (183.0–248.5)	0.71	206.00 (152.50–256.00)	223.00 (183.00–248.00)	0.414
Prothrombin time (seconds), median (IQR)	11.2 (10.7–12.0)	11.3 (10.7–12.12)	0.513	11.50 (10.75–12.15)	11.30 (10.70–11.90)	0.425
International normalized ratio, median (IQR)	1.03 (0.97–1.09)	1.04 (0.97–1.11)	0.499	1.04 (0.98–1.10)	1.03 (0.97–1.10)	0.516
Activated partial thromboplastin time (seconds), median (IQR)	29.6 (27.08–32.42)	29.1 (26.43–33.32)	0.886	29.40 (27.10–31.85)	29.10 (26.55–33.25)	0.92
ssICH location, *n* (%)			0.77			0.461
Deep	168 (93.33)	51 (94.44)		46 (90.20)	48 (94.12)	
Lobar	12 (6.67)	3 (5.56)		5 (9.80)	3 (5.88)	
Presence of intraventricular hemorrhage, *n* (%)	67 (37.22)	19 (35.19)	0.785	16 (31.37)	18 (35.29)	0.674
Hematoma expansion, *n* (%)	14 (7.78)	13 (24.07)	0.001	4 (7.84)	12 (23.53)	0.029
Surgical treatment, *n* (%)	15 (8.33)	5 (9.26)	0.831	4 (7.84)	4 (7.84)	1.00
mRS score, *n* (%)			0.001			0.003
0–2	101 (56.11)	17 (31.48)		32 (62.75)	17 (33.33)	
3–6	79 (43.89)	37 (68.52)		19 (37.25)	34 (66.67)	

ssICH, spontaneous supratentorial intracerebral hemorrhage; mRS, modified Rankin scale; SD, standard deviation; IQR, interquartile range.

**Figure 4:**
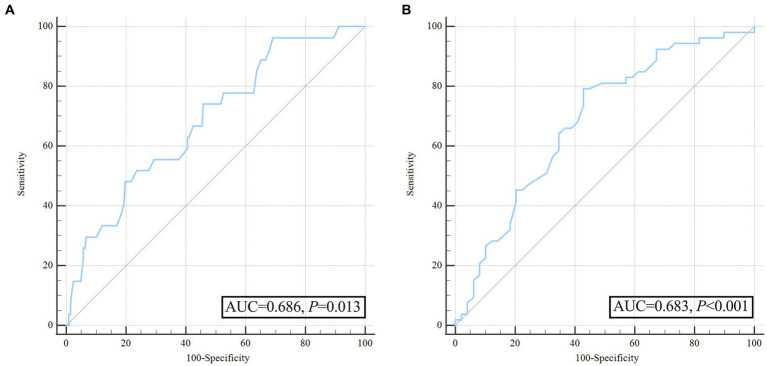
Receiver operating characteristic curve analysis of pulse pressure for predicting hematoma expansion and unfavorable outcome after propensity score matching. **(A)** ROC for pulse pressure to predict hematoma expansion after propensity score matching; **(B)** ROC for pulse pressure to predict poor outcome after propensity score matching.

To further account for significant differences in baseline characteristics, we conducted a one-to-one PSM. Age, sex, hypertension, smoking, diabetes mellitus, alcoholism, baseline ssICH volume, admission GCS score, time from onset to initial CT, hemoglobin, platelets, PT, INR, aPTT, ssICH location, and presence of IVH were included in PSM. After adjusting for potential confounding with PSM (caliper 0.01, ratio 1:1, nearest neighbor approach), early HE events (*p* = 0.029) and poor outcome (*p* = 0.003) still had statistically significant differences between the two groups (Table 5). The incidence of HE events and unfavorable outcomes remained significantly higher in the group with wider pulse pressure, as evidenced by the statistical analysis (HE, 12/51[23.53%] vs. 4/51[7.84%], *p* = 0.029; poor outcome, 34/51[66.67%] vs. 19/51 (37.25%), *p* = 0.003; Table 5). According to the ROC curve, the AUC of pulse pressure level for predicting early HE and poor prognosis was 0.686 and 0.683, respectively (Figures 4A,B; Table 3).

## Discussion

The present study investigated the association between pulse pressure and early HE in patients with ssICH. Our results demonstrated that widened acute pulse pressure (≥98 mmHg) levels upon admission are associated with increased risks of early hematoma expansion in patients with ssICH. Moreover, a significant link exists between admission pulse pressure levels and an increased risk of early HE in the multivariate logistic regression analysis, weighted generalized additive model, and RCS models. Lastly, using a 1:1 PSM to balance the variations in baseline characteristics, we found that admission pulse pressure remained an independent predictor for early hematoma enlargement and unfavorable prognosis. To our knowledge, this was the first study to investigate the association of admission pulse pressure with early HE using nonlinear models.

sICH is commonly attributed to acute hypertension and prolonged high BP can worsen the secondary injury. It can lead to early hematoma enlargement by fueling the active hemorrhagic process in the acute phase and exacerbating tissue and microvascular damage in the subsequent hours. Despite the lack of detriment to rapid BP control shown in the ATACH2 study (22), it failed to demonstrate any benefits for preventing HE or improving functional outcomes. A prior study by Larse and colleagues illustrated that elevated prehospital DBP and MAP correlated with HE in sICH patients (19). Still, there was no link between prehospital pulse pressure and HE in their study (19). However, our study is inconsistent with this study. We found that wider pulse pressure levels upon admission are associated with increased risks of early HE in ssICH patients. First, the background of the patients studied by Larse et al. differed from ours. The monitoring of BP was performed in a prehospital ambulance in his research, and BP measurements may be inaccurate (as mentioned by the authors in Limitations) (19). Our BP monitoring was monitored at a steady state upon admission. Furthermore, the previous study encompassed all hematoma locations, including lobar, deep, infratentorial, and unclassifiable ICH, while our research specifically focused on patients with ssICH.

Wide pulse pressure can be visualized in the classic Cushing’s response to elevated intracranial pressure (25). The Cushing triad typically includes bradycardia, respiratory depression, and hypertension, which may be associated with brainstem distortion or ischemia. Hence, the link between wide pulse pressure and more severe findings on admission CT scans (such as larger hematoma volume and significant midline shift) may be attributed to a pathophysiological reflex activated by elevated intracranial pressure (18). The present study highlighted an association between elevated pulse pressure at admission and an increased likelihood of early HE during hospitalization. Even though we observed the correlation, the underlying mechanisms driving the connection between increased pulse pressure and HE in ssICH patients remain elusive. The potential underlying mechanism might be as follows. First, wider pulse pressure imposes increased mechanical stress on the vascular walls, particularly in the microvasculature surrounding the site of intracranial hemorrhage. This heightened stress may compromise the integrity of endothelial cells and the basal membrane, potentially contributing to the initiation of early HE (26). Second, increased pulse pressure may disrupt the blood–brain barrier. This disruption allows components from the bloodstream to permeate into the brain tissue, initiating inflammatory responses and exacerbating neuronal damage, thereby promoting hematoma growth (27, 28). Third, high pulse pressure levels could lead to autonomic nervous system dysregulation, impairing blood flow autoregulation. This imbalance might contribute to hematoma enlargement, especially within the microvascular network near the site of intracranial hemorrhage (29). Fourth, elevated pulse pressure may subject the vascular walls to mechanical stress, inducing structural alterations and the formation of micro-fissures. This pathway could provide a route for further hematoma expansion by compromising the structural integrity of blood vessels (30). Fifth, increased pulse pressure might trigger inflammatory responses, accelerating HE by migrating inflammatory cells and releasing inflammatory mediators. The complex network of inflammatory reactions could contribute to the observed association (31). Sixth, elevated pulse pressure levels may increase the propensity for hemorrhagic transformation, creating an environment conducive to early HE in ssICH patients (32). The association between increased pulse pressure and heightened risk of early HE in ssICH patients involves a multifaceted interplay of vascular, inflammatory, and autoregulatory mechanisms. A deeper understanding of these mechanisms provides a foundation for developing targeted interventions to mitigate early HE and improve patients’ prognosis. Further research into these intricate pathways holds promise for optimizing treatment strategies and advancing personalized approaches to the acute management of ssICH.

Our study demonstrated that 27 patients (11.54%) experienced early HE events, indicating a decreased incidence of hematoma enlargement compared to our previous reports (21, 24). Upon analysis, we determined the potential reasons as follows. Our last study encompassed all sICH, including deep, lobar, brainstem, and cerebellum, whereas the current study focused on ssICH. Second, based on the latest literature (19), the present study defined the time point for follow-up CT as 25 h, which is different from the time point of the previous study (24 h) (24). Thirdly, the current study did not exclude patients with an initial hematoma volume of <1 mL. The initial ICH hematoma volume in this study was markedly smaller compared to that in the previous study (8. 4 [4.16–14.0] mL. vs.11.95 [5.0–28.64] mL). Additionally, over the last 3 years, a few sICH patients with more critical conditions were hospitalized in the neurointensive care unit (NICU) at our hospital, yet they were not included in the present study.

The current study revealed that wider pulse pressure (≥78 mmHg) was an independent predictor for poor prognosis based on ROC curve. Interestingly, our study differs from the results of Yu Hua Huang et al. (18), who contended that pulse pressure over 100 mmHg was not correlated with a poor prognosis but solely with mortality during hospitalization. According to the study by Yu Hua Huang et al., a pulse pressure value greater than 100 mmHg was identified as a risk factor for cardiovascular disease prognosis and mortality (25). However, this measurement may not accurately represent the pulse pressure situation of intracranial lesions,especially in patients with ICH induced by hypertension. Additionally, Yu Hua Huang et al. defined mRS 4–6 as a poor diagnosis, whereas we defined mRS 3–6 as a poor diagnosis based on the more common previous literature (23). This difference in mRS definitions may also explain the different conclusions reached.

Several limitations existed in the present study. Firstly, inherent in the design of our single-center, retrospective, observational study with a relatively small sample size is an inability to conclude causation. Additionally, we did not incorporate patients with ssICH in the NICU, who were more critically ill, potentially causing bias. Our strict exclusion criteria may limit the applicability of our findings to other clinical presentations, such as brain stem hemorrhage and infratentorial hematomas, which are often included in general ICH literature. Thirdly, our study failed to account for potential confounders, such as shifts in blood pressure management and treatment strategies during hospitalization, which might have influenced the outcomes. A single admission BP value is not representative of all BP variability. The study’s robustness would have been greatly improved if ambulatory BP monitoring had been utilized. Fourthly, the associations between BP and imaging features of early HE, such as the CT island sign and CT angiographic spot sign, were not analyzed, as previous similar studies had already covered this topic (33). Finally, despite our study finding a notable link between pulse pressure and early HE, the precise mechanism driving this correlation is still not fully understood. Additional research is required to elucidate the mechanisms and explore potential therapeutic interventions that target pulse pressure.

## Conclusion

Widened acute pulse pressure (≥98 mmHg) levels upon admission are associated with increased risks of early hematoma expansion in patients with ssICH. This observation underscores the importance of measuring pulse pressure in future trials investigating the association between BP reduction and HE in ssICH patients. Nevertheless, multi-center, larger-sample prospective studies are needed to verify the conclusions further, and basic research is warranted to elucidate the mechanisms.

## Data availability statement

The original contributions presented in the study are included in the article/Supplementary material, further inquiries can be directed to the corresponding authors.

## Ethics statement

The studies involving humans were approved by Medical Ethics Committee of Dehua County Hospital. The studies were conducted in accordance with the local legislation and institutional requirements. Written informed consent for participation was not required from the participants or the participants’ legal guardians/next of kin because this was a retrospective study and informed consent was not required according to Chinese law.

## Author contributions

C-YW: Conceptualization, Data curation, Investigation, Software, Visualization, Writing – original draft, Writing – review & editing. S-ZL: Data curation, Investigation, Methodology, Writing – original draft. B-CK: Data curation, Methodology, Visualization, Writing – original draft. Y-ZL: Data curation, Formal Analysis, Methodology, Writing – original draft. C-JC: Data curation, Investigation, Resources, Visualization, Writing – original draft. X-BH: Conceptualization, Data curation, Methodology, Writing – original draft. J-QW: Formal Analysis, Supervision, Writing – original draft, Writing – review & editing.
