# Supplementary material for: Association of pulse pressure with hematoma expansion in patients with spontaneous supratentorial intracerebral hemorrhage
Source: Front Neurol. 2024 May 15;15:1374198. doi: 10.3389/fneur.2024.1374198 (PMC11133623; doi:10.3389/fneur.2024.1374198)
Supplement: Supplementary file 1 [file Table_1.docx]

Table S1 The Shapiro–Wilk normality test for continuous variables.

| Characteristics | *P*-Value |
| --- | --- |
| Age | 0.002 |
| Systolic blood pressure | 0.372 |
| Diastolic blood pressure | <0.001 |
| Mean arterial pressure | 0.053 |
| Pulse pressure | 0.093 |
| Time from symptom onset to initial CT | <0.001 |
| Admission GCS score | <0.001 |
| Baseline ssICH volume(ml), median | <0.001 |
| Hemoglobin | 0.033 |
| Platelets | <0.001 |
| Prothrombin time | <0.001 |
| International normalized ratio | <0.001 |
| Activated partial thromboplastin time | <0.001 |

CT, computed tomography; GCS, Glasgow Coma Scale; ssICH: spontaneous supratentorial intracerebral hemorrhage.
